# Dressing for the Open Air

**Published:** 1891-05

**Authors:** 


### DRESSING FOR THE OPEN AIR.

There is one thing that mothers and nurses often do thoughtlessly, and with serious results to the children. This is the habit of dressing them for their out-of-door drive or walk in the warm nursery, and then letting them stand around for some time while the nurse gets herself ready. For that is the usual practice. The nurse dresses the baby first, even to gloves and tippet, and then she begins leisurely on the details of her own toilet. Meanwhile the little one waits about in the warm room, getting warmer and warmer, until at last, when it gets into the outer air, it is in a perspiration that will induce a chill at once. A child should not have its wraps put on until the very last moment. And then it should be done, not in a warm room, but in the hall, from which it should be carried as soon as the operation is completed.

